# Correlation of Vitamin D deficiency with chest X-rays severity scores and different inflammatory markers in severe and critical COVID-19 patients

**DOI:** 10.4314/gmj.v56i4.3

**Published:** 2022-12

**Authors:** Sajjad Ali, Ashraf, Nabi Rahman, Jamal Nasir, Nelofer Akbar

**Affiliations:** 1 Mardan Medical Complex Medical Teaching Institution Bacha Khan Medical College, Pakistan; 2 Pro-Gene Diagnostic And Research Laboratory, Pakistan

**Keywords:** Vitamin D Deficiency, COVID-19, Chest X-Ray, Inflammatory Markers, Severe, Critical

## Abstract

**Objective:**

To determine the relationship between Vitamin D deficiency with Chest X-Rays severity score and Different Inflammatory Markers in Severe and Critical COVID-19 Patients.

**Design:**

A cross-sectional study

**Setting:**

The study was conducted in COVID-19 isolation units at Mardan Medical Complex Teaching Hospital (MMCTH) and Bacha Khan Medical College, Pakistan

**Participants:**

206 patients who tested positive for COVID-19 by PCR were included in the final analysis.

**Data Collection/Intervention:**

We collected demographic, comorbidity, laboratory, and clinical outcome data from the electronic records of admitted, deceased, or discharged patients.

**Main outcome measure:**

Frequency of symptoms, comorbidities, mortality and morbidity, chest x-ray severity scores, different inflammatory markers in Vitamin D deficient Covid-19 patients

**Results:**

128(62.14%) were severe and 78(37.5%) were critical COVID-19 patients. The whole cohort had 82(39.80%) males and 124(60.20%) females, with a median age of 55 IQR (50-73). Study participants' median Vitamin D level was 14.01ng/ml, with a minimum of 7.5ng/ml and a maximum of 70.8ng/ml. 67/206 patients died, with a fatality ratio of 32.5%. 54/67(80.59%) suffered from one or more comorbid conditions.

**Conclusion:**

Low Vitamin D levels were linked to a higher risk of death, higher x-ray severity scores, and different inflammatory markers. Vitamin D levels greater than 30ng/ml for older patients and greater than 40ng/ml in older patients with comorbidities were associated with reduced severity and mortality in patients with COVID-19.

**Funding:**

None declared

## Introduction

Vitamin D boosts the innate immune response, lowering the risk of infection from SARS-CoV-2 exposure. Vitamin D is also involved in regulating the adaptive immune system and inflammation. Infected people with sufficient levels are less likely to develop hyperinflammatory (severe) COVID-19 (the cytokine or bradykinin “storm”).^1^ Vitamin D suppresses viral multiplication by releasing defensin and cathelicidin proteins from macrophages and monocytes.^2^,^3^ To eliminate respiratory pathogens, Vitamin D induces apoptosis and autophagy in the affected epithelium.^4^ Some COVID-19 patients with severe symptoms had low T-lymphocyte counts.^5^ Vitamin D supplementation boosts T-lymphocyte levels 6, supporting the idea that it could help cure COVID-19 or reduce the severity and progression to mortality.

One of the pandemic's most serious issues is severe COVID-19 progression in some patients. Thrombotic events and cytokine storms have been linked to severe COVID-19 infection. These events cause fatalities.^7^–^8^ Vitamin D reduces cytokine storm risk and controls thrombotic pathways. 9,10 Vitamin D deficiency has been directly linked to increased COVID-19 severity and mortality. 11,12

In recent years, Vitamin D deficiency has increased the risk of multiple illnesses, including systemic infection.^13^ Since Vitamin D plays an immunomodulation role^14^, production of antiviral peptides to enhance innate immunity^3^ and bolstering mucosal defenses, Vitamin D deficiency can impact immunological activities. In clinical investigations, acute respiratory illnesses, such as influenza epidemics, have been linked to low serum Vitamin D levels.^15^,^16^ In a recent meta-analysis including data from different observational studies^17^, Serum Vitamin D levels of <20 ng/ml were linked to a 64% higher risk of community-acquired pneumonia. Vitamin D deficiency has been suggested in recent reviews to reduce lung immune function, hence raising the risk of COVID-19 severity and death.^18^,^19^

SARS-CoV-2 is rapidly spreading and poses an unpredictable risk to human health worldwide.^20^ Acute respiratory disease syndrome (ARDS)^21^ appears to be caused by SARS-CoV-2 predominantly through the immune evasion mechanism during infection, followed by hyper response and cytokine storm in some individuals.^22^ Angiotensin-converting enzyme 2 allows SARS-CoV-2 to infiltrate alveolar and intestinal epithelial cells at the cellular level.^24^ When the reninangiotensin system is out of whack, it might cause an influx of cytokines, which can eventually cause lethal acute respiratory distress syndrome.^23^

As a result of these findings, the impact of Vitamin D deficiency on COVID-19 infection and outcomes has gathered substantial attention. Studies on the association between Vitamin D deficiency and COVID-19 mortality are scarce, and most current research focuses on COVID-19 infection, severity, and therapy. So, the literature on this topic calls for more clinical investigations. 24,25,26

It is crucial to learn why COVID-19 is so different in terms of mortality and severity worldwide. Better nutrition's potential to boost immunity is a significant consideration. Vitamin D and other nutrients play essential roles in immune system function. However, Vitamin D's potential function in reducing COVID-19 infection and mortality is little understood. Therefore, this study aimed to determine if there is a correlation between Vitamin D deficiency with chest x-ray severity scores and inflammatory marker levels in severely and critically ill COVID-19 patients.

## Methods

This cross-sectional study was conducted in the COVID-19 isolation units of the Mardan Medical Complex (MMC) in Khyber Pakhtunkhwa, Pakistan, from November9, 2021, to March21, 2022. The sample was raised using the convenience sampling technique. We collected demographic, comorbidity, laboratory, and clinical outcome data from the electronic records and the charts of deceased or discharged admitted patients. COVID-19 patients in this study were split into two groups: Severe and Critical.

Respiratory distress equivalent to 30 breaths per minute, oxygen saturation <93% at room air, arterial oxygen partial pressure (PaO2), or FiO2 of 300mmHg corresponding to 0.133kPa) were considered “severe” in COVID-19 patients. As determined by an independent medical practitioner, if a patient needed mechanical ventilation, was in septic shock, or required to be admitted to an intensive care unit, they were considered “critical” (ICU).^27^

### Inclusion and exclusion criteria

Adult patients over the age of 18 years who tested positive for COVID-19 by rRT-PCR and were classified as severe or critical COVID-19 patients were included in the final analysis after providing written informed consent within the first 24 hours of admission. Patients who tested negative on rRT-PCR for COVID-19, patients below the age of 18 years, patients referred to another medical facility during hospitalization, patients hospitalized more than once, and patients whose Vitamin D test results were found missing were excluded.

### Inflammatory markers and Vitamin D measurements

HORIBA ABX micros es60 hematology analyzer, Roach Cobas e411 Immunoassay Analyzer, and an Automated Chemistry Analyzer Architect c4000 abbot were used to measure hemoglobin, total leukocytes count, lymphocytes count, neutrophils count, platelets count, D-Dimer, C-Reactive Protein, Ferritin, Lactate Dehydrogenase, and Vitamin D. Many guidelines define Vitamin D deficiency as serum Vitamin D levels <20ng/mL. The Mayo Clinic's definition of Vitamin D insufficiency (<30ng/ml) was used in this study.^28^

### Chest X-Ray Severity Scores criteria

The chest X-ray severity scores were calculated based on chest x-ray involvement using the RALE score. Each radiograph was divided into four quadrants (Q1, Q2, Q3, Q4) by the vertebral column vertically and the left major bronchus initial branch horizontally. Each quadrant was given a score from 0–4 (0 = no involvement; 1=25%; 2=25%–50%; 3=50%–75% 4=>75%) depending on the degree of interaction. Visible opacity was graded 1–3. (density score) (1=hazy, 2=moderate, 3=dense) if an independent or blind investigator score is assigned, rale scores range from 0 (no infiltrates) to 48 (dense consolidation in >75 % of each quadrant).^29^

### Ethical considerations

Patients were enrolled in this study after authorization by Institutional Review Boards Reference No 192/BKMC, Dated 01/11/2021, of Mardan Medical Complex and Bacha Khan Medical College Pakistan. Participants gave their fully informed consent and were free to opt-in or out of the study at any point.

Participants were required to sign the box showing their informed consent, confirming that they had read the research purpose, understood it, and wanted to participate. To acquire a clear image of the study, uneducated participants were instructed in their native tongue about its significance. All participants received assurances of confidentiality, and all collected data were treated with confidentiality.

### Statistical analysis

Data are presented as appropriate, as the median and interquartile range (IQR), or as frequencies and percentages. To explore the connection between categorical factors such as gender, age, comorbidities, symptoms, and chest radiography, the Pearson Chi-Square Test and Fisher Exact Test were utilized. The Kruskal Wallis One-Way Analysis of Variance (ANOVA) was used to test for inter-group differences in severe patients, critical patients, and Vitamin D deficiency. The 95% confidence interval was used for all statistical tests (95%CI). The Pearson Correlation Method was used to investigate the correlation between Vitamin D Deficiency with Chest X-ray severity scores and different inflammatory Markers. Relationships between Vitamin D Deficiency with Chest X-ray severity scores and different inflammatory Markers were studied using scatter plots. SPSS version 26 was used for all statistical analyses. P-value <0.05 was considered statistically significant.

## Results

### Demographic characteristics and symptoms

A total of 243 patients were examined, and in the final analysis, 206 patients were included who met the inclusion criteria. 37 COVID-19 patients without Vitamin D test results were excluded. 128(62.14%) were severe patients, and 78(37.85%) were critical patients. Of the patients in this study, 82(39.80%) were males, and 124(60.20%) were females. The median age was 55, with an interquartile range of 50-73. Symptoms included fever 206(100%), sore throat 115(55.82%), shortness of breath 188(91.26%), chest pain 107(51.94%), fatigue 134(65.04%), myalgia 157(76.21%) and loss of test and smell 157(76.21%), whereas 0(0.00%) were asymptomatic. Comorbid conditions included diabetes 26(12.62%), hypertension 32(15.53%), Asthma 28(13.59%) Hepatitis C 5(2.42%), Hepatitis B 3(1.45%), COPD 42(20.38%), cancer 1(0. 48%).(Table 1)

**Table 1:** Demographics and Patient Characteristics

Demographic charact	Whole Cohort	Severe	Critical	P-value
Total Patients	206(100)	128(62.14)	78(37.86)	
Gender				
Male	82(39.80)	45(35.15)	37(47.43)	0.10
Female	124(60.20)	83(64.85)	41(52.57)	0.14
Age	55(50–73)	60(50–65)	61(54–73)	0.006
Min	21	21	25	
Max	85	74	85	
Patients Characteristics				
Symptoms				
Asymptomatic	0(0.00)	0(0.00)	0(0.00)	***
Fever	206(100)	128(62.14)	78(37.86)	***
Sore Throat	115(55.82)	56(43.75)	59(75.64)	0.00
Shortness of Breath	188(91.26)	112(87.5)	76(97.43)	0.20
Chest pain	107(51.94)	39(30.46)	68(87.17)	0.00
Fatigue	134(65.04)	81(63.28)	53(67.94)	0.54
Myalgia	157(76.21)	96(75.00)	61(78.20)	0.73
loss of Smell and Test	152(73.78)	79(61.71)	73(93.58)	0.00
Comorbidities				
Diabetes	26(12.62)	15(11.71)	11(14.10)	0.66
Hypertension	32(15.53)	16(12.5)	16(20.51)	0.16
Asthma	28(13.59)	09(7.00)	19(24.35)	0.001
Hepatitis C	05(2.42)	00(0.00)	05(6.41)	0.007
Hepatitis B	03(1.45)	01(0.78)	02(2.56)	0.55
COPD	42(20.38)	16(8.98)	26(33.33)	0.001
Cancer	01(0.48)	01(0.78)	0(0.00)	1.00

Total Patients, Gender, symptom, and comorbidities are expressed as total number and percentage. While individual Age data is in the form of Median (IQR). Min=Minimum Test Range/Max=Maximum Test Range. P-values *** indicate that no statistics were computed because either the characteristic was present in all or none of the patients. P-value <0.05 is statistically significant.

### Vitamin D levels of Severe and Critical COVID-19 patients

This study's median Vitamin D level was 14.01ng/ml, with an interquartile range of 11.6ng/ml to 25.77ng/ml, a minimum of 7.5ng/ml, and a maximum of 70.8ng/ml. The median Vitamin D level for patients with severe illness was 20.90ng/ml, with an interquartile range of 14.87 to 24.47ng/ml, a minimum of 8.6 and a maximum of 70.8ng/ml. Critical patients' median Vitamin D level was 16.88ng/ml, with a minimum of 7.5ng/ml and a maximum of 49.2ng/ml. 28(21.87%) severe patients had Vitamin D levels <10ng/ml, 26(20.13%) had levels <20ng/ml, 44(34.37%) had levels <30ng/ml, and 30(23.43%) had levels >30ng/ml. 19(24.35%) of the critical patients had Vitamin D levels <10ng/ml, 24(30.76%) had levels <20ng/ml, 23(29.48%) had levels <30ng/ml, and 12(15.38%) had levels >30ng/ml. (Tables 2,3 and 4)

**Table 2:** Inflammatory markers and Chest X-Ray involvement score characteristics

Characteristics	Whole cohort	Severe	Critical	P value
**Screening Markers**				
**Hemoglobin**	12.1(11.4–13.1)	12.9(11.4–13.0)	12.1(11.3–13.8.)	0.001*
**Min**	10.1	10.3	9.8	
**Max**	15.9	14.6	15.9	
**Total Leukocyte Count**	9.9(8.2–11.5)	9.4(8.0–11.3)	13.8(10.3–16.0)	0.001*
**Min**	6.11	6.3	9.3	
**Max**	36.9	13.5	36	
**Lymphocytes**	4.6(2.9–7.8)	6.2(4.0–8.52)	2.65(1.67–6.82)	0.001*
**Min**	0.15	2.4	0.15	
**Max**	29.3	29.3	10	
**Neutrophils**	89.4(83.9–92.3)	86.1(82.2–90.0)	91.8(90.3–94.0)	0.001*
**Min**	71.6	71.6	80	
**Max**	98	95.7	98	
**Platelets Count**	304(263–327)	292(275–232)	314(253–359)	0.041
**Min**	112	166	112	
**Max**	501	395	501	
**Inflammatory Markers**				
**Lactate Dehydrogenase**	708(473–959)	667(473–827)	893(470–1147)	0.001
**Min**	178	178	114	
**Max**	1951	1100	1927	
**C-Reactive Protein**	22.4(15.6–31.2)	16.5(11.5–21.57)	33.15(29–38.0)	0.001
**Min**	0.11	0.11	22	
**Max**	77.4	27.4	77.4	
**Ferritin**	1509(895–1894)	714(473–1413)	1892(1610–2164)	0.001*
**Min**	36	36	69	
**Max**	4312	2694	4312	
**D-Dimer**	847(482–1443)	673(401–831)	1586(1084–1989)	0.000
**Min**	191	191	266	
**Max**	10944	1837	10944	
**Vitamin D**	14.01(11.61–25.77)	20.90(14.87–24.47)	16.88(11.19–22.96)	0.001*
**Min**	7.5	8.6	7.5	
**Max**	70.8	70.8	49.2	
**Chest X-Ray Involvement Score**				
**0:No Involvement**	0(0.00)	0(0.00)	0(0.00)	
**1:≤25%**	0(0.00)	0(0.00)	0(0.00)	
**2:25–50%**	0(0.00)	0(0.00)	0(0.00)	
**3:50–75%**	139(67.47)	112(62.92)	27(34.61)	0.001
**4:≥75%**	67(32.52)	16(12.5)	51(65.38)	0.003

Screening and Inflammatory Markers are in the form of Median (IQR) while Total Patients and Chest X-Ray data is expressed as total number and percentage.

Min=Minimum Test Range/Max=Maximum Test Range

Hemoglobin Normal: Male:13.2–16.6gm/dl, Female:11.6–15gm/dl, Total Leukocyte Count Normal:4.0–11.0,103/ul, Lymphocytes Normal:20–40%, Neutrophils Normal:40–60 %, Platelets Count Normal:150–400 106/mm3, Lactate Dehydrogenase Normal:140–280u/l, C-Reactive Protein Normal:0–10mg/l, Ferritin Normal: Male:27–250, Female:20–140ng/ml D-Dimer Normal:100–250 ng/ml, Vitamin D: Normal:>30 ng/ml

P-value <0.05 is statistically significant.

* p-value=<0.001

**Table 3:** Multivariable association of Vitamin-D deficiency with Severe Covid-19 patients

Characteristics	Severe	Vitamin D Deficiency: ng/ml						
		<10	<20	<30	>30	P-value	95%cl	P-value
Total Patients	128(62.14)	28(21.87)	26(20.31)	44(34.37)	30(23.43)			
Screening Markers								
Hemoglobin	12.9(11.4–13.0)	11.0(10.5–11.3)	11.5(11.3–11.9)	12.6(12.2–13.0)	13.7(12.8–14.2)	0.001*	12.0–12.4	0.000
Min	10.3	10.1	10.9	12	12.6			
Max	14.6	11.5	12	13.5	14.6			
Total Leukocyte Count	9.4(8.0–11.3)	12.1(11.3–13.0)	11.0(10.1–11.6)	8.3(8.0–8.4)	7.5(6.6–8.0)	0.001*	9.1–9.7	0.000
Min	6.3	10.9	9.8	7.9	6.3			
Max	13.5	13.51	11.9	8.7	8.3			
Lymphocytes	6.2(4.0–8.52)	3.6(2.9–1.3)	3.9(3.4–4.6)	6.4(5.4–7.8)	9.9(9.3–11.1)	0.001*	5.7–6.7	0.000
Min	2.4	2.4	2.9	4.1	8			
Max	29.3	4.6	4.9	29.3	12.3			
Neutrophils	86.1(82.2–90.0)	92.6(90.2–94.3)	88.5(87.4–91.1)	84.7(81.8–87.4)	81.0(75.8–84.1)	0.001*	85.2–87.1	0.000
Min	71.6	89	85.1	81	71.6			
Max	95.7	95.7	92.5	91	85.5			
Platelets Count	292(275–232)	298(251–327)	314(298–323)	310(299–327)	251(211–283)	0.001*	284–299	0.000
Min	166	205	289	166	185			
Max	395	372	395	327	283			
Inflammatory Markers								
Lactate Dehydrogenase	667(473–827)	952(868–1058)	776(700–847)	618(515–723)	364(296–438)	0.001*	627–707	0.000
Min	178	868	672	442	181			
Max	1100	1092	1100	828	489			
C-Reactive Protein	16.5(11.5–21.57)	23.0(20.6–25.8)	21.2(20.1–22.9)	15.5(12.2–18.4)	7.8(3.1–11.4)	0.001*	15.3–17.7	0.000
Min	0.11	19	18.2	8.4	3.17			
Max	27.4	27	23.9	24	11.4			
Ferritin	714(473–1413)	2075(1741–2385)	1544(1287–1701)	915(598–1156)	560(405–742)	0.001	1056–1279	0.000
Min	36	1429	1101	104	250			
Max	2694	2681	1847	1493	799			
D-Dimer	673(401–831)	1286(1037–1594)	542(441–630)	397(311–494)	251(211–283)	0.003	604–741	0.000
Min	191	895	383	191	198			
Max	1837	1837	751	651	296			
Chest X-Ray Involvement Score								
0:No Involvement	0(0.00)	0(0.00)	0(0.00)	0(0.00)	0(0.00)			
1:≤25%	0(0.00)	0(0.00)	0(0.00)	0(0.00)	0(0.00)			
2:25–50%	0(0.00)	0(0.00)	0(0.00)	0(0.00)	0(0.00)			
3:50–75%	112(62.92)	19(67.85)	22(84.61)	37(84.09)	26(86.66)	0.002		
4:≥75%	16(12.5)	9(32.15)	4(15.38)	7(15.91)	4(13.34)	0.005		

Screening and Inflammatory Markers are in the form of Median (IQR) while Total Patients and Chest X-Ray data is expressed as total number and percentage.

Min=Minimum Test Range/Max=Maximum Test Range

Hemoglobin Normal: Male:13.2–16.6gm/dl, Female:11.6–15gm/dl, Total Leukocyte Count Normal:4.0–11.0,103/ul, Lymphocytes Normal:20–40%, Neutrophils Normal:40–60 %, Platelets Count Normal:150–400 106/mm3, Lactate Dehydrogenase Normal:140–280u/l, C-Reactive Protein Normal:0–10mg/l, Ferritin Normal: Male:27–250, Female:20–140ng/ml D-Dimer Normal:100–250 ng/ml, Vitamin D: Normal:>30 ng/ml

P-value <0.05 is statistically significant.

* p-value=<0.001

**Table 4:** Multivariable association of Vitamin-D deficiency with Critical Covid-19 Patients

Characteristics	Critical	Vitamin D Deficiency: ng/ml						
		<10	<20	<30	>30	P-value	95%Cl	P-value
Total Patients	78(37.86)	19(24.35)	24(30.76)	23(29.48)	12(15.38)			
Screening Markers								
Hemoglobin	12.1(11.3–13.8.)	11.3(10.3–11.9)	12.6(11.6–13.0)	12.7(12.7–14.4)	13.9(13.1–15.02)	0.001*	12.0–12.7	0.000
Min	9.8	9.8	11	10.7	13.17			
Max	15.9	12	14.2	15.9	14.4			
Total Leukocyte Count	13.8(10.3–16.0)	12.7(11.6–14.1)	10.95(10.1–12.7)	9.5(8.8–11.1)	8.7(7.6–9.3)	0.001*	10.3–11.2	0.000
Min	9.3	11	10.1	6.2	7			
Max	36	15	36.9	11.1	9.9			
Lymphocytes	2.65(1.67–6.82)	1.9(1.0–2.4)	1.5(0.85–2.3)	6.2(4.6–7.4)	8.9(7.1–9.4)	0.001*	3.2–4.6	0.000
Min	0.15	0.4	0.1	2.4	6.2			
Max	10	2.7	3	8	10			
Neutrophils	91.8(90.3–94.0)	95.7(95.1–97.1)	92.7(91.9–93.3)	90.6(90.3–91.2)	82.7(81.1–83.7)	0.001*	90.3–92.3	0.000
Min	80	92.2	91.2	89.6	80			
Max	98	94	98	92	85			
Platelets Count	314(253–359)	357(317–464)	313(262–342)	311(245–368)	165(144–259)	0.012	289–326	0.000
Min	112	300	313	236	112			
Max	501	501	342	393	289			
Inflammatory Markers								
Lactate Dehydrogenase	893(470–1147)	1602(1431–1831)	1168(921–1390)	370(228–552)	467(337–662)	0.000	824–1070	0.000
Min	114	1255	870	114	266			
Max	1927	1927	1586	691	798			
C-Reactive Protein	33.15(29–38.0)	47.2(35.0–53.90	32.7(28.8–38.47)	32.5(29.4–33.7)	30.5(28.6–34.5)	0.000	33.2–36.8	0.000
Min	22	31.3	27.8	26.4	28			
Max	77.4	59.1	77.4	34.9	36.3			
Ferritin	1892(1610–2164)	2497(2216–2730)	1978(1792–2115)	1726(1597–1824)	1087(905–1429)	0.003	1771–1995	0.000
Min	69	2032	1508	1508	69			
Max	4312	3061	4321	2431	1501			
D-Dimer	1586(1084–1989)	3098(2574–3626)	1835(1639–1907)	1092(957–1176)	899(844–1073)	0.009	1561–1952	0.000
Min	266	2063	1508	827	802			
Max	10944	10944	1965	1290	1192			
Chest X-Ray Involvement Score								
0:No Involvement	0(0.00)	0(0.00)	0(0.00)	0(0.00)	0(0.00)			
1:≤25%	0(0.00)	0(0.00)	0(0.00)	0(0.00)	0(0.00)			
2:25–50%	0(0.00)	0(0.00)	0(0.00)	0(0.00)	0(0.00)			
3:50–75%	19(24.35)	3(15.78)	5(20.83)	5(21.73)	6(50.0)	0.003		
4:≥75%	59(75.64)	16(84.22)	19(79.16)	18(78.26)	6(50.0)	0.007		

Screening and Inflammatory Markers are in the form of Median (IQR) while Total Patients and Chest X-Ray data is expressed as total number and percentage.

Min=Minimum Test Range/Max=Maximum Test Range

Hemoglobin Normal: Male:13.2–16.6gm/dl, Female:11.6–15gm/dl, Total Leukocyte Count Normal:4.0–11.0cells/cm, Lymphocytes Normal:20–40%, Neutrophils Normal:40–60 %, Platelets Count Normal:150–400 106/mm3, Lactate Dehydrogenase Normal:140–280u/l, C-Reactive Protein Normal:0–10mg/l, Ferritin Normal: Male:27–250, Female:20–140ng/ml D-Dimer Normal:100–250 ng/ml, Vitamin D: Normal:>30 ng/ml

P-value <0.05 is statistically significant.

* p-value=<0.001

### Inflammatory markers of Severe and Critical COVID-19 patients

In COVID-19 patients, the inflammatory marker values in the severe group with Vitamin D levels <30ng/ml were considerably higher than those in the severe group with Vitamin D levels >30ng/ml, showing a significant difference between the two groups. In the critical group, the inflammatory markers were much greater than in the severe group of vitamin D levels <30ng/ml vs >30ng/ml. When comparing severely ill and critically ill COVID-19 patients, however, both groups of patients revealed statistically significant evidence that Vitamin D levels >30ng/ml reduced the severity of inflammatory markers in COVID-19 patients. (Table 3 and 4)

### Chest X-Ray Severity Scores of Severe and Critical COVID-19 patients

The total Chest X-ray severity scores were 128(62.14%) in the severe group and 78(37.86%) in the critical group. The number of Chest X-ray severity scores was lowest in critical COVID-19 patients, with 12(15.38%) in >30ng/ml Vitamin D group compared to severe COVID-19 patients, 30(23.43%) had the lowest >30ng/ml Vitamin D group. In critical COVID-19 patients, the number of patients increased by 66(84.61%) when <30ng/ml vitamin levels were employed compared to severe COVID-19 patients when <30ng/ml vitamin levels were used, 98(76.56%) patients had Vitamin D levels <30ng/ml. Thus, Vitamin D levels of >30ng/ml reduced the chest X-ray severity scores in patients diagnosed with COVID-19. (Table 3,4)

### Correlation between Vitamin D deficiency with inflammatory marker and chest X-ray Severity Scores

**Figure 3:**
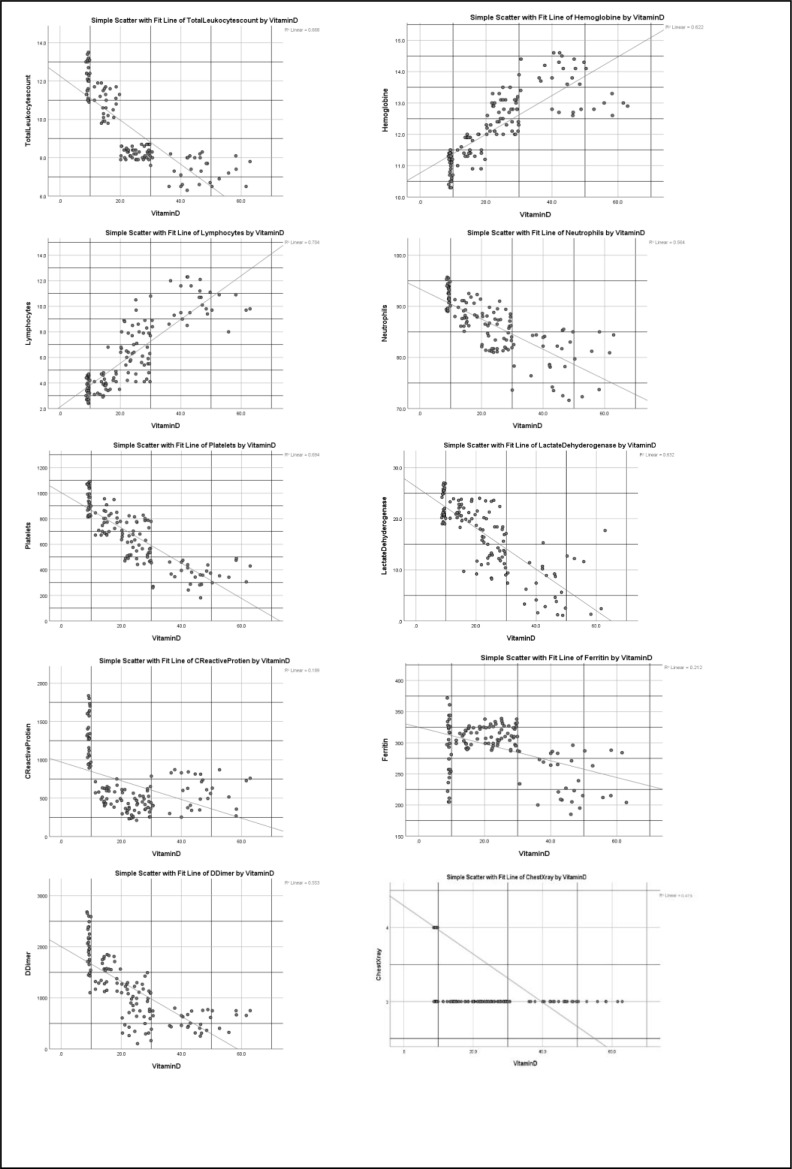
Scatter plots show the relationship between vitamin D deficiency and different inflammatory markers and chest X-ray severity scores in severe COVID-19 patients.

**Figure 4:**
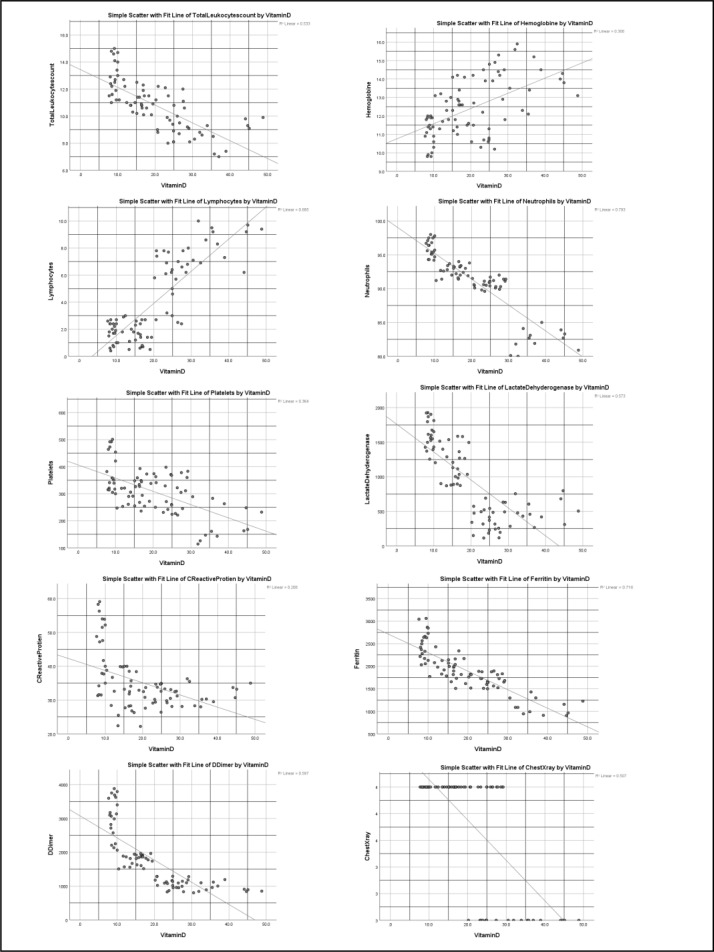
Scatter plots show the relationship between vitamin D deficiency and different inflammatory markers and chest X-ray severity score in critical COVID-19 patients.

A substantial positive and robust negative association was found in the Pearson correlation and scatter plot between Vitamin D and chest X-ray severity score and inflammatory markers. Vitamin D had a high positive correlation with hemoglobin and lymphocytes in severe and critical patients but a negative correlation with total leukocyte count, neutrophils, Platelets, C-reactive protein, Lactate dehydrogenase, D-dimer, ferritin, and chest X-ray Rale score. (Table 5, Figures 3 and 4)

**Table 5:** Correlation of Vitamin D deficiency with screening markers, different inflammatory markers, and chest X-ray involvement score

Characteristics	Severe Patients		Critical Patients	
	Correlation Coefficient	P-value	Correlation Coefficient	P-value
Screening Markers				
Haemoglobin	0.789**	0.000	0.548**	0.000
Total Leukocyte Count	-0.817**	0.000	-0.730**	0.000
Lymphocytes	0.753**	0.000	0.828**	0.000
Neutrophils	-0.751**	0.000	-0.890**	0.000
Platelets Count	-0.460**	0.000	-0.603**	0.000
Inflammatory Markers				
Lactate Dehydrogenase	-0.833**	0.000	-0.757**	0.000
C-Reactive Protein	-0.795**	0.000	-0.656**	0.000
Ferritin	-0.744**	0.000	-0.846**	0.000
D-Dimer	-0.434**	0.000	-0.774**	0.000
Chest X-Ray Involvement Score				
0:No Involvement				
1:≤25%				
2:25–50%				
3:50–75%	-0.822**	0.000	-0.769**	0.000
4:≥75%	-0.713**	0.000	-0.753**	0.000

** Correlation is significant at the 0.01 level (2-tailed).

### Deceased COVID-19 patients' Vitamin D levels

**Figure 1:**
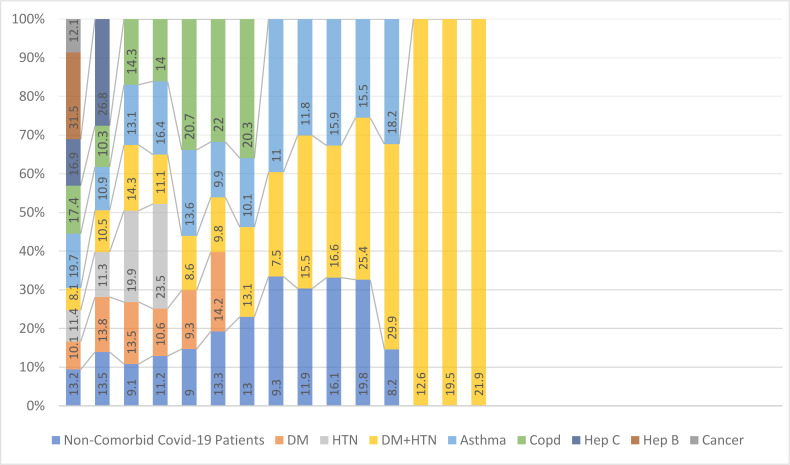
A case-to-case representation of deceased patients with their vitamin D levels. 67 out of 206 (32.52%) patients died at severe and critical stage of COVID -19 infection. 54 out of 67 (80.59%) patients died were comorbid patients.

A total of 67/206 patients died with a fatality ratio of (32.52%). Of the deceased, 54/67(80.59%) suffered from one or more comorbid conditions, whereas the remaining 13 were non-comorbid. A deceased patient had Vitamin D levels of a minimum of 7.5ng/ml and a maximum of 31.5ng/ml. (Figure1)

## Discussion

In this study, patients with Vitamin D deficiency, particularly severe and critical patients, as well as older age and with multiple comorbidities, had a higher risk of death from COVID-19 infection. Because higher Vitamin D levels were found to have a strong negative relationship with worse chest x-ray severity scores and excess inflammation as measured by inflammatory markers, adequate Vitamin D levels may protect the immune system, particularly in the elderly. In addition, Vitamin D seemed to mute the disadvantages of having multiple comorbidities in severe and critical COVID-19 patients. Our findings suggest that Vitamin D deficiency may increase the incidence of COVID-19 severity and its progression to mortality.

This study's favourable results concerning Vitamin D sufficiency being somewhat protective in COVID-19 are consistent with earlier findings. Receiving some sun is probably required to recuperate from Vitamin D insufficiency. Total sun hours from March to April were 333.4 in 2019 and 349.4 in 2020. The difference in sunlight was only 4.8%, which is insignificant; however, Vitamin D supplementation and dietary habits are likely to be key issues.^30^ The role of Vitamin D during the COVID-19 pandemic in reducing infection risk, disease severity, and death was fiercely disputed.^31^ Just as India has a high rate of Vitamin D deficiency,^32^ 52.91% of COVID-19 patients in Pakistan had Vitamin D levels of <20ng/ml in our study. The proportion was 79.11% when a <30ng/mL limit was employed to evaluate Vitamin D deficiency levels.

Kernan, K. *et al.* found that ferritin is a significant immune dysregulation mediator through its direct immunosuppressive and pro-inflammatory activities, particularly in severe hyperferritinemia. An essential host defensive mechanism during infection is elevated ferritin levels, which remove defence immune cell function. Additionally, it could be protective by reducing free radical generation and regulating immunomodulation.^33^ In a study from Spain, Vitamin D insufficiency was found to dramatically boost ferritin levels, which was likewise observed in our study.^34^ An Indian study compared previously published data on average Vitamin D levels with death reports from multiple states and concluded that Death rates might be more significant in Vitamin D deficient areas. However, this study had substantial limitations because individual Vitamin D levels were not tested, and most of the historical data used was heterogeneous and limited.^32^ Our findings are consistent with other hospital-based studies, which found that low Vitamin D levels were linked to severe/critical COVID-19 disease,^35^,^36^ greater rates of ICU admission,^37^ and higher levels of inflammatory markers,^38^ and death.^33^An interesting finding of this study^39^ was that Vitamin D insufficiency is associated with increased elderly morbidity.

In this study's Severe and Critically COVID-19 patients, there appears to be a relationship between Vitamin D deficiency and high D-dimer levels. Elevated D-dimer levels signal the activation of pro-inflammatory cytokine cascades (and downregulation of the anti-inflammatory cytokine cascade). When D-dimer levels are high, the risk of death increases.^40^

The cytokine storm observed in COVID-19 infection may be due to Vitamin D deficiency-induced dysregulation of innate and adaptive immunity. Vitamin D has an impact on both bacterial and viral innate immune responses. It works by inhibiting pro-inflammatory cytokines includes such as interleukin (IL)-1, interleukin (IL)-6, interleukin (IL)-8, interleukin (IL)-12, and tumor necrosis factor-alpha TNF-α.^41^ Vitamin D stimulates the development of T regulatory cells while inhibiting the transition of naive T cells into pro-inflammatory Th17 cells.^42^,^43^ Vitamin D has anti-inflammatory effects in human alveolar epithelial cells and helps wound healing.^44^ Vitamin D also helps maintain the endothelium's health, and a lack of it causes vascular permeability and leakage.^45^–^46^ Men are more vulnerable to ACE2 receptor dysregulation and, presumably, higher COVID-19 morbidity due to Vitamin D deficiency since it increases the X-chromosome-associated “Renin-Angiotensin” System (RAS) activity.^47^

Vitamin D and CRP had a -0.879 correlation value, which was significant in inflammatory disorders (p=0.001).^48^ taking COVID-19 into account as an inflammatory condition. Our study found a correlation coefficient of -0.795 with a p-value=0.000 for severe patients and a correlation coefficient of -0.656 for critical patients. Multiple connections between lymphocyte immunological markers and Vitamin D insufficiency were discovered in individuals with CRVD, CAD, T2DM, and hypertension.^49^ Our study found associations between lymphocyte inflammatory markers and Vitamin D deficiency, such as COVID-19 patients who showed higher percentages of severe and critical cases and were more likely to die.

Older COVID-19 patients with comorbidities were more likely to indicate low immune function, with higher rates of comorbidities including diabetes, hypertension, asthma, COPD, hepatitis B, hepatitis C, and cancer. This was supported by the proportion of patients with a decreased number of lymphocytes and an increase in inflammatory markers in older COVID-19 patients being significantly higher than that in non-comorbid COVID-19 patients. Pimental *et al.* found that neutrophil counts increased in the low Vitamin D group compared to the normal Vitamin D group^50^ and our findings were comparable.

Campi *et al.* found that Vitamin D levels were significantly lower in patients admitted to ICU with COVID-19, as were LDH and platelet levels.^51^ Our investigation found that Vitamin D deficiency was also associated with lower LDH and platelet levels in COVID-19 patients. In addition, this study discovered a positive relationship between Vitamin D deficiency and hemoglobin level, consistent with previous studies demonstrating that Vitamin D deficiency with low hemoglobin level and rachitic symptoms were associated with increased severity of acute lower respiratory tract infections.^52^

Waheed, S. et al. found that Chest X-ray at presentation showed bilateral ground-glass appearance in Vitamin D-deficient COVID-19 patients. After three days of Vitamin D supplementation, it showed improvement of chest X-ray ground-glass appearance.^53^ In another study, Breslin É. et al. found that low Vitamin D levels were associated with a higher risk of infiltrates on chest X-rays.^54^ in our investigation, a significant negative correlation between chest X-ray severity scores and Vitamin D deficiency in severe and critical COVID-19 patients, were discovered.

The conclusions of the current study have several limitations. First, the investigation was done in a single centre (Mardan, Pakistan) multi-centre study would provide more strong conclusions. Second, From November 2021 to April 2022, less sun exposure, a direct source of Vitamin D, may aggravate the illness. Despite this limitation, winter seasonal variation demonstrates that this study population was significantly less exposed to sunlight. In addition, because this study only included individuals with low sun exposure, these findings may not apply to those with high sun exposure. Third, only quantitative indicators were measured, with no regard for the time between infection and admission. This information was unavailable at the time of admission. The time between infection and admission is a significant independent predictor of infection risk. In COVID-19 patients, timely hospitalization has a significant beneficial effect on reducing severity progression to mortality.

Fourth, when evaluating inflammatory symptoms, individuals with asymptomatic, mild, or moderate presentations were advised to stay at home and were not included in the current study. Our findings show that Vitamin D deficiency is not common in these patients, so the majority of patients admitted to the hospital were in a severe and critical stage of the disease, were deficient in Vitamin D, and linked to an increased incidence of COVID-19 hospitalization. Fifth, most of our patients were elderly, an independent risk factor for COVID-19. It is suggested that countries in which elderly people live among the general population should impose more severe preventive mitigation measures than countries in which elderly people are kept apart from the general population. This is due to the fact that the disease spreads more quickly and severely among elderly patients with Vitamin D deficiency than it does among younger patients.

## Conclusion

Vitamin D modulates the immune system in addition to improving the ability of the innate immune system to combat COVID-19 infection. Vitamin D is also involved in the regulation of the adaptive immune system and inflammation. Vitamin D deficiency was correlated with an increase in several inflammatory markers and chest x-ray severity scores. In Severe and Critical COVID-19, Vitamin D levels greater than 30ng/ml in older age and greater than 40ng/ml in older patients with comorbidities were associated with reduced severity and mortality. Randomised controlled trials and large population research should be conducted to validate these preliminary findings.

## Figures and Tables

**Figure 2:**
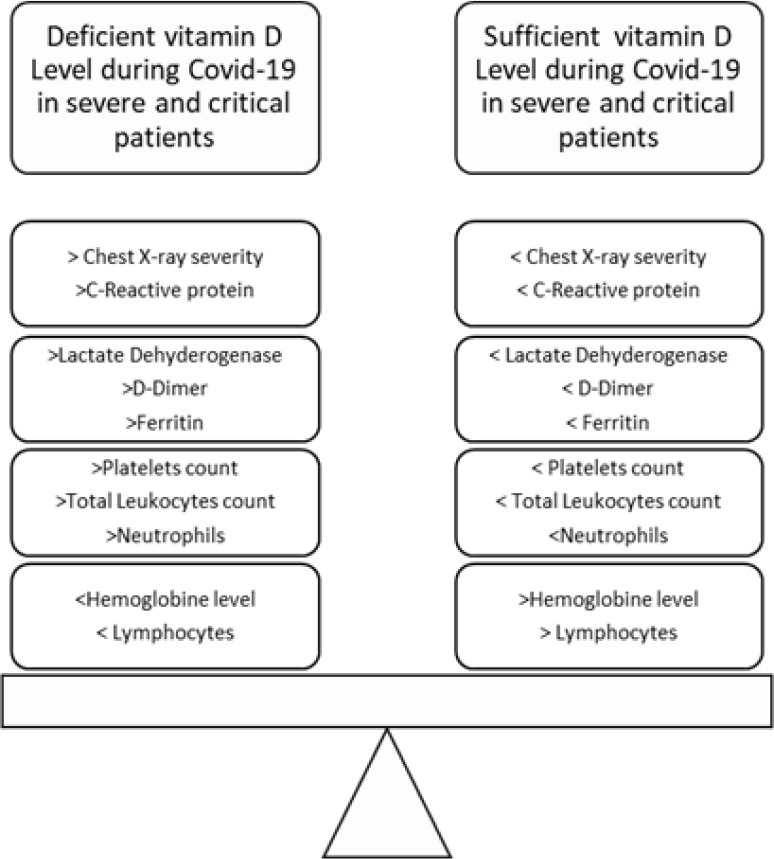
A summary-based result from this study.
